# Influence of the Metallic Sublayer on Corrosion Resistance in Hanks’ Solution of 316L Stainless Steel Coated with Diamond-like Carbon

**DOI:** 10.3390/ma17184487

**Published:** 2024-09-12

**Authors:** Ewa Dobruchowska, Justyna Schulz, Viktor Zavaleyev, Jan Walkowicz, Tomasz Suszko, Bogdan Warcholinski

**Affiliations:** Faculty of Mechanical Engineering and Energetics, Koszalin University of Technology, Sniadeckich 2, 75-453 Koszalin, Poland; ewa.dobruchowska@tu.koszalin.pl (E.D.); justynaschulz@interia.pl (J.S.); zavaleyev@gmail.com (V.Z.); jan.walkowicz@tu.koszalin.pl (J.W.); tomasz.suszko@tu.koszalin.pl (T.S.)

**Keywords:** diamond-like carbon coatings, metallic adhesive sublayers, pulsed vacuum-arc evaporation, corrosion resistance, adhesion

## Abstract

The purpose of the study was to ascertain the corrosion resistance in Hanks’ solution of Cr-Ni-Mo stainless steel (AISI 316L) coated with diamond-like carbon (DLC) coatings to establish its suitability for biomedical applications, e.g., as temporary implants. The influence of the carbon coating thickness as well as the correlated effect of the metallic sublayer type and defects present in DLC films on corrosion propagation were discussed. The results obtained were compared with findings on the adhesion of DLC to the steel substrate. The synthesis of carbon thin films with Cr and Ti adhesive sublayers was performed using a combined DC and a high-power-impulse vacuum-arc process. Evaluation of the corrosion resistance was carried out by means of potentiodynamic polarisation tests and scanning electron microscopy. Adhesive properties of the sublayer/DLC coating systems were measured using a scratch tester. It was found that systems with Ti sublayers were less susceptible to the corrosion processes, particularly to pitting. The best anti-corrosion properties were obtained by merging Ti with a DLC coating with a thickness equal to 0.5 μm. The protective properties of the Cr/DLC systems were independent of the carbon coating thickness. On the other hand, the DLC coatings with the Cr sublayer showed better adhesion to the substrate.

## 1. Introduction

The growing demands placed on metallic biomaterials, utilised in orthopaedic implantology and dental engineering, necessitate the development of new materials and the refinement of existing ones to ensure optimal performance. Currently, Cr-Ni-Mo stainless steels and Co-Cr-Mo and Ti-Al-V alloys are the most commonly used metallic biomaterials for temporary implants and long-term endoprostheses [1,2,3,4]. Austenitic stainless steels, in particular, are in high demand due to their low cost and ease of production [1]. All of these alloys display satisfactory mechanical and physico-chemical properties. However, when used in a biologically active environment, endoprostheses are susceptible to fatigue processes, as well as corrosion and frictional wear [5,6]. The released ions and insoluble wear products deposit in the space between the implant and the bone, thereby enhancing damage to the endoprosthesis until it becomes loose. Furthermore, the accumulation of metal ions in the soft tissues adjacent to the joint has the potential to negatively impact the biological homeostasis [7,8].

The chemical stability and biocompatibility of metallic implants can be enhanced by modifying their surface (surface treatment). The current solutions, which also encompass medical steels, include low-temperature plasma nitriding and carburising [9,10], the deposition of diamond-like carbon (DLC) coatings [11,12] and the deposition of ceramic coatings that are primarily based on Ti, Zr, and Ta oxides [13,14,15]. Among the aforementioned protective coatings, those comprising DLC films continue to be of interest due to the combination of their properties, which are highly desirable in the context of biomedical applications [16]. These materials exhibit high hardness, high friction wear resistance, electrical insulation, chemical inertness, and good biocompatibility [17]. The existing literature on the biotolerance of carbon-based coatings primarily concerns the following materials: hydrogenated amorphous carbon (a-C:H), tetrahedral amorphous carbon (ta-C), and carbon nitride (CN) [18,19,20]. Nevertheless, ta-C films with a high concentration of sp^3^ bonds, as a consequence of their optimal tribological characteristics, are recommended for use in knee and hip replacements (e.g., artificial femoral heads)—implants that operate in particularly challenging conditions [20,21].

In this work, the 316L austenitic stainless steel coated with thin carbon films, characterised by a high content of tetrahedral amorphous carbon phase and high hardness [22], was the object of the study. Given that the thickness of the coating influences the distribution of internal stresses within the film and, thus, its continuity, two different thicknesses of DLC coatings were subjected to testing. Another factor that affects the protective properties of DLC coatings is their adhesion to the substrate. The adhesion of the coating can be enhanced by the use of metallic sublayers that contribute to the reduction of stress within the overall substrate/carbon coating system [23,24]. The adhesive layers are composed of metals from the transition groups of the periodic system, with Cr being the primary component [22,25]. However, in biological systems, chromium (in metallic form or as Cr ions) may exert cytotoxic effects and trigger allergic hypersensitivity towards metal ions [6]. It is also important to note that pores are often present in the DLC coating structure, even in cases of high quality [26], which enables body fluids to contact the sublayer and/or the substrate. In light of these considerations, it is evident that the substitution of Cr with alternative metals/compounds that have a less harmful effect on the human body and its homeostatic mechanisms is of significant importance. Therefore, in addition to the 316L stainless steel coated with Cr/DLC, tests were also conducted on systems comprising Ti sublayers. Ti is renowned for its passive behaviour towards the biological environment of the human body, as well as for its proclivity to form a stable TiO_2_ layer, which is responsible for its exceptional corrosion resistance [27].

The primary objective of the research was to determine the corrosion resistance of the substrate/metallic sublayer/DLC systems in a simulated body fluid environment (Hanks’ solution). Two metals were selected as materials for the sublayers: Cr, which is regarded as the most suitable in terms of adhesion, and Ti, which exhibits excellent biocompatibility. Although the corrosion behaviour of various DLC-coated alloys has been the subject of numerous studies, the correlated effect of the type of metallic sublayer and the defects (pores) present in carbon coatings of different thicknesses on local corrosion propagation has not been widely discussed. A comparative analysis was therefore conducted on the porosity (degree of defectivity) of DLC coatings deposited on two different sublayers and the electrochemical behaviour of the aforementioned sublayers in contact with the electrolyte within the defects (i.e., their ability to undergo passivation and repassivation processes), as well as on the adhesion of metallic sublayer/DLC coating assemblies to 316L austenitic steel. The expected results will provide guidance for the design and manufacture of DLC (ta-C) coatings to improve the corrosion resistance of temporary austenitic stainless steel implants.

## 2. Materials and Methods

### 2.1. Sample Preparation

The synthesis of DLC thin films with Cr and Ti adhesion sublayers was carried out using direct-current vacuum-arc deposition with superimposed high-current arc pulses, as described in [22]. The experiments were conducted in the C55CT industrial vacuum-arc device manufactured by INOVAP GmbH. Pure Cr and Ti cathodes were employed to deposit metallic sublayers with a thickness of 0.1 µm. The thickness of all the coatings was measured with the Calotest method (spherical abrasion test). Cathodes made of hot-pressed graphite with a purity of 99.999% (Kurt J. Lesker GmbH, Dresden, Germany) and a diameter of 70 mm were utilised in the DLC deposition experiments. Two 50 A DC arc cathodes were superimposed with 1400 A high-current pulses, as described by [28], at frequency of 100 Hz and for a 300 μs duration. Argon gas (purity 99.999%) was used to enhance the operational stability of the vacuum-arc plasma sources. No filtering system was applied, and a deposition system diagram has been published elsewhere [22].

Prior to deposition, stainless steel (AISI 316L; chemical composition detailed in Table 1) disc-shaped substrates, with dimensions of 30 mm × 3 mm, were polished to a roughness of *R*a = 0.02 µm. Subsequently, the specimens were subjected to ultrasonic cleaning in acetone and alcohol, after which they were mounted on the substrate holder within the vacuum chamber that had been evacuated to a pressure of 1 × 10^−3^ Pa. The temperature was controlled by a thermocouple that was in direct contact with the substrate. In light of the findings of our preceding studies [22], the substrate temperature of 90 °C was selected as a means of achieving a balance between the compressive stress within the film and the ratio of the sp^3^ bonds to the sum of the sp^2^ and sp^3^ bonds. A range of 56–58% was assumed for the ta-C fraction. The estimated hardness of the coatings thus obtained equates to approximately 40 GPa [22].

The detailed procedure and parameters of the deposition processes of the Me (Cr or Ti)/DLC systems are shown in Table 2. The duration and number of steps in the DLC deposition stage were adjusted to achieve a total thickness of the resulting carbon coatings of 0.2–0.25 μm (6 steps) and 0.5 μm (12 steps) for the samples designated 316L/Me/0.2DLC (316L/Cr/0.2DLC, 316L/Ti/0.2DLC) and 316L/Me/0.5DLC (316L/Cr/0.5DLC, 316L/Ti/0.5DLC), respectively.

### 2.2. Sample Characterisation

Although DLC coatings, being amorphous, do not produce a diffraction pattern, due to the nature of the substrate and the metal sublayers used, the samples were subjected to X-ray diffraction testing. The study was conducted using a Panalytical Empyrean 3 diffractometer (Malvern Panalytical Ltd., Malvern, UK) with Cu-Kα radiation. Two configurations were employed in this study: a Bragg–Brentano (BB) configuration and a configuration with a glancing angle (GA) ω = 3°. In the first case, the tilt angle ψ was set to 0, resulting in the acquisition of interference reflections from planes parallel to the sample surface. The second configuration exhibited a reduced depth of X-ray penetration. By comparing the diffraction patterns, the preferred crystallographic orientation with respect to the sample surface can be observed.

To assess corrosion resistance of the 316L steel substrates coated with Me (Cr or Ti) sublayer/DLC systems, potentiodynamic polarisation tests were performed using the Atlas 0531 Electrochemical Unit (Atlas Sollich, Gdansk, Poland). The measurements were conducted in a conventional three-electrode cell at room temperature (25 ± 1 °C) in an environment of simulated body fluid–Hanks’ solution (Sigma-Aldrich Chemie GmbH, Steinheim, Germany, pH = 7.22) with the chemical composition given in Table 3. The volume of the solution used for each test was 0.03 dm^3^, and the surface area of the samples (working electrodes) exposed to its action was 0.29 cm^2^. A saturated standard calomel electrode (SCE, Hg/Hg_2_Cl_2_/KCl) and a platinum plate were used as the reference and counter electrodes, respectively. Prior to commencing the polarisation tests, the samples were left to interact with the corrosive medium for 1 h under open-circuit conditions. The measurements were conducted at a scan rate of 0.001 V/s within a potential range from −0.6 V/SCE to 1.3 V/SCE and were repeated up to three times for each sample. The representative polarisation curves have been subsequently used to estimate the corrosion potential (*E*_corr_) and the corrosion current density (*i*_corr_) by the Tafel extrapolation method [29]. The Stern–Geary equation was used to calculate the polarisation resistance (*R*_p_) [30]. The *R*_p_ values were then used to estimate the porosity of the sublayer/coating systems according to [31,32]. Cyclic potentiodynamic polarisation measurements were taken, starting from the corrosion potential and scanning towards higher potentials until *E* reached the value of 0.400 V/SCE (or the anodic current density was 5 × 10^−3^ A/cm^2^). The direction of the scan was then reversed. In addition to the polarisation tests, open-circuit potential (OCP) changes were recorded during the 12 h immersion of the samples in Hanks’ solution.

Uncorroded and corroded parts of the samples were examined by scanning electron microscope (SEM, FEI Quanta 200 Mark II (FEI s.r.o., Brno, Czech Republic)—pristine samples; LV 5500, JEOL (JEOL Ltd., Tokyo, Japan)—corroded samples). The SEM observations were made in a vacuum environment at a pressure of 2 × 10^−5^ Pa and an electron beam acceleration voltage of 20 kV. In addition, the chemical composition of selected samples was analysed using an EDAX Genesis XM 2i X-ray microanalyser (EDAX Inc., Mahwah, NJ, USA) retrofitted to an FEI Quanta 200 Mark II scanning electron microscope.

Adhesive properties of the metallic sublayer/DLC coating systems were measured using the scratch tester REVETEST^®^ (CSM Instruments, Peseux, Switzerland) equipped with a diamond type C Rockwell indenter with a radius of 200 μm. During the measurements, the indenter moved at a velocity of 10 mm/min, and the loading force increased linearly from 0.9 N to 50 N.

## 3. Results and Discussion

**Figure 1:**
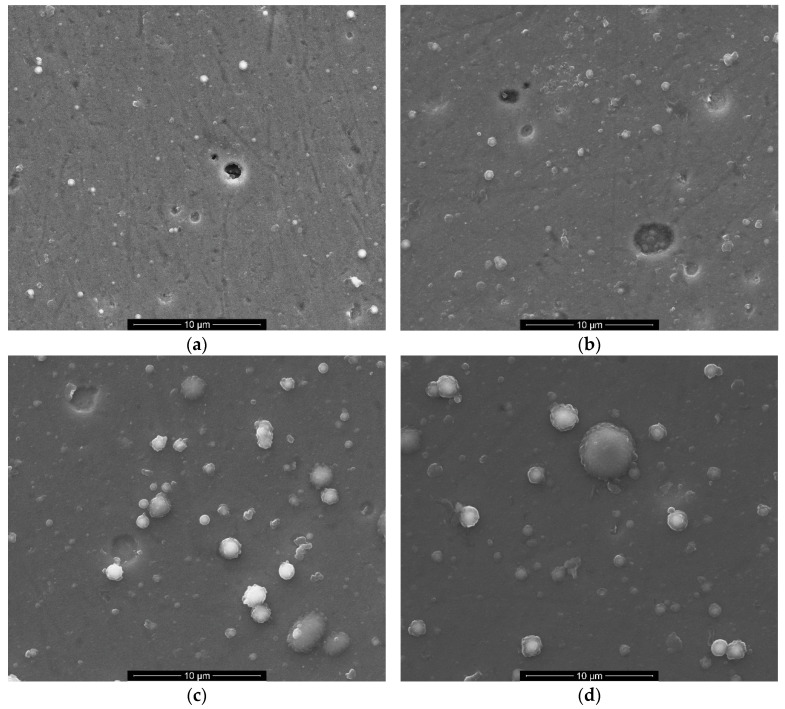
SEM images of the surface of as-deposited samples: 316L/Cr/0.2DLC (**a**), 316L/Cr/0.5DLC (**b**), 316L/Ti/0.2DLC (**c**), and 316L/Ti/0.5DLC (**d**).

The SEM micrographs revealed a multitude of defects on the surface of all the samples tested, irrespective of the type of sublayer employed and the thickness of the carbon coating. The 316L/Cr/DLC systems (Figure 1a,b) are distinguished by the presence of numerous pits, craters, and pinholes. In the case of the 316L/Cr/0.2DLC specimen, these features can extend through the entire thickness of the coating, thus exposing the Cr sublayer. Defects in the coating may also include droplets deposited on the surface. These droplets are closely related to the process of arc evaporation of the cathode material [33]. The droplets decorating the surface of the 316L/Cr/0.2DLC and 316L/Cr/0.5DLC samples are spherical in shape and limited to tenths of a micron in size.

A slightly different morphology is observed for DLC coatings deposited on Ti sublayers, as shown in Figure 1c,d. The microdroplets are more numerous and exhibit greater variation in size. The diameter of the largest of them is ca. 4 μm. Additionally, irregularly shaped particles are visible, which are likely the result of the agglomeration of particles ejected from the cathode before reaching the coating surface [33]. X-ray microanalysis demonstrated that carbon was the predominant constituent of the droplets/particles. Microdroplets larger than 1 µm are less firmly bonded to coatings and are more easily extracted [34], as demonstrated by the presence of circular voids on the surface of the 316L/Ti/0.2DLC sample. However, no evidence was found of defects that affected the coating’s continuity.

**Figure 2:**
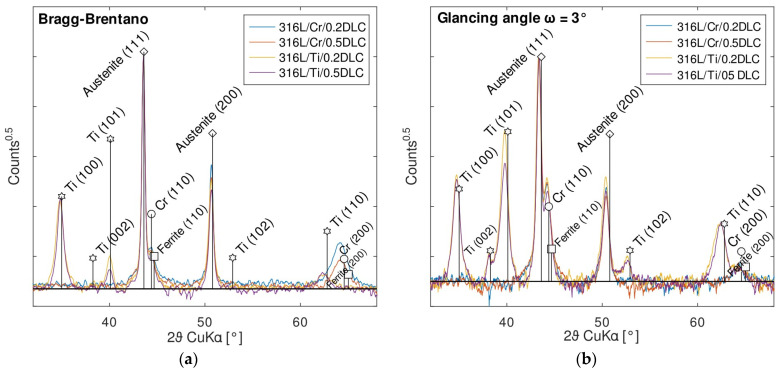
X-ray diffraction patterns of the samples under investigation obtained in the Bragg–Brentano configuration (**a**) and in a configuration with a small constant angle of incidence, i.e., ω = 3° (**b**).

The X-ray diffraction patterns recorded for the 316L/Cr/DLC and 316L/Ti/DLC samples are presented in Figure 2. The results of this study permit a number of observations to be made. 

The peak at 44.5° can be interpreted as either deriving from the substrate or the Cr sublayer. However, it is more likely to originate from the substrate (most probably from ferrite formed during surface grinding), because this peak is almost identical in each sample, as are the peaks at angles of 43.6° and 50.7°, which clearly originate from austenitic substrates (ICDD card No. 04-019-2390). The only chromium (ICDD 00-006-0694) peak visible in the XRD pattern is (200), observed at 64.2°. This peak was recorded in a Bragg–Brentano configuration, indicating that the Cr sublayers exhibit a strong orientation with planes of the (200) family parallel to the substrate surface.

This texture also appears on the substrates with Ti sublayers. The proportions of peak intensities in the patterns recorded in the GA configuration are broadly consistent with those reported in the ICDD database (card No. 00-071-0860). However, in the case of the diffractograms recorded in the BB geometry, the dominance of the (100) peak is evident.

Differences in the position of the peaks originating from the substrates in the two diffractometer configurations can also be seen: (1) GA-(111) 43.4°, (200) 50.4°; (2) BB-(111) 43.6°, (200) 50.65°. Larger diffraction angles in the BB configuration (smaller interplane spacings) indicate tensile stresses in the substrates parallel to their surfaces.

On the other hand, no Cr carbide or Ti carbide peaks are observed, which may be related to the reduction of the substrate temperature to 90 °C [22] prior to carbon deposition. It is known that during the deposition process, a transition zone is formed between the metallic sublayer and the DLC coating, which may contain different carbide phases depending on the zone formation temperature. Our previous studies on Cr/DLC systems have shown that at a substrate temperature of 195 °C, Cr_23_C_6_ carbides are formed, which adversely affect the adhesion of the DLC coatings to the steel substrate. The scratch test results also showed that the adhesion of the DLC coatings increased as the substrate temperature decreased [22]. The absence of metal carbides is therefore also expected to be a factor in improving the corrosion resistance of 316L/Me/DLC systems (Me = Cr or Ti).

**Figure 3:**
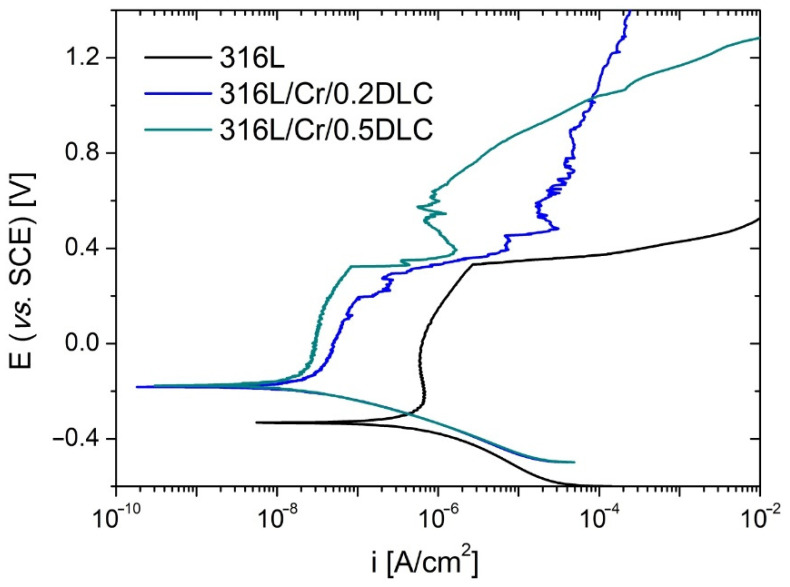
Polarisation curves obtained for uncoated and Cr/DLC-coated 316L steel substrates in Hanks’ solution.

**Figure 4:**
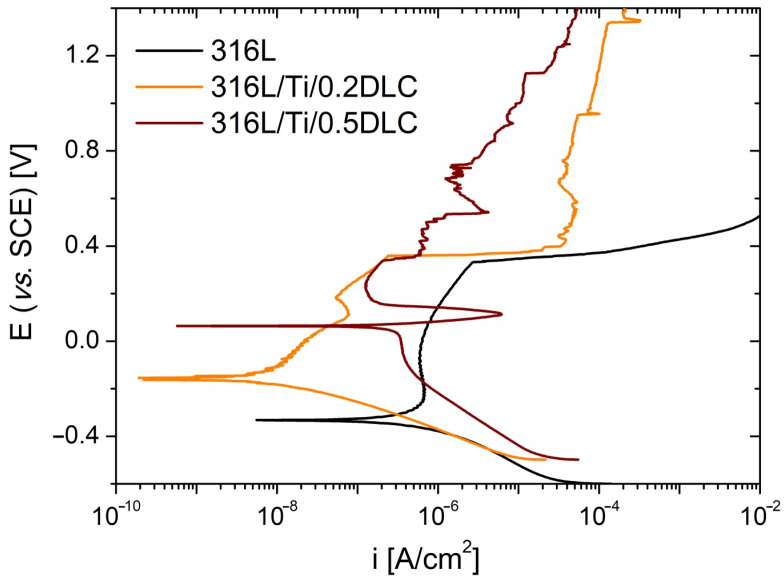
Polarisation curves obtained for uncoated and Ti/DLC-coated 316L steel substrates in Hanks’ solution.

The electrochemical corrosion resistance of 316L stainless steel coated with Cr/DLC and Ti/DLC was evaluated in comparison to the bare steel substrate. The potentiodynamic polarisation curves recorded in Hanks’ solution for the samples with Cr and Ti sublayers are shown in Figure 3 and Figure 4, respectively, while the electrochemical parameters characterising the corrosion processes are collated in Table 4.

An analysis of the polarisation curve obtained for the uncoated steel (depicted in black in Figure 3 and Figure 4) reveals the presence of three distinct anodic regions. These observations align with those reported by other authors in electrochemical studies conducted on 316L in various chloride-rich media [35,36,37]. They correspond to (1) dissolution of the alloying constituents, which lasts until approximately *E* = −0.220 V/SCE; (2) active–passive transition; and (3) an unstable passive region, which is observed until the breakdown potential (*E*_b_) value of 0.331 V/SCE is reached. The occurrence of the plateau within the third region is attributed to the formation of the passive layer on the steel surface, which consists of the oxidation products of alloying elements, mainly Cr (III) oxide, although the presence of Mo^4+^ and Mo^6+^ cannot be excluded [38,39]. A portion of Cr_2_O_3_ is oxidized in an aqueous environment to produce Cr(OH)_3_. The transition at *E*_b_ is followed by a break in passivity and a sharp increase in anodic current density associated with the formation of stable pits [40].

The polarisation curves obtained for 316L/Cr/DLC systems, although similar in shape to those recorded for bare steel, are characterised by higher values of corrosion potential (Figure 3 and Table 4). This behaviour reflects a higher corrosion resistance of the samples in the Hanks’ solution environment. An increase in *E*_corr_ is associated with a decrease in corrosion current density and an increase in polarisation resistance (Table 4). The *i*_corr_, according to Faraday’s law, is the electrochemical corrosion rate. The *R*_p_, measured near the corrosion potential, has a similar meaning and is inversely proportional to *i*_corr_, according to the Stern–Geary equation, where *b*_a_ and *b*_c_ are the anodic and cathodic Tafel slopes, respectively [30]:

(1) $$i_{\mathrm{corr}}=\frac{b_{a}b_{c}}{2.303R_{p}(b_{a}+b_{c})}.$$

Both of these quantities can therefore be considered measures of the metal oxidation reaction rate in the corrosive environment used. The values of *i*_corr_ and *R*_p_ estimated for both 316L/Cr/DLC systems indicated a significant slowing of the corrosion kinetics compared with those for the uncoated steel. However, these effects are almost independent of the thickness of the DLC layer. The similar behaviour of 316L/Cr/0.2DLC and 316L/Cr/0.5DLC suggests a discontinuity in the DLC component. This observation is consistent with SEM images of the surface morphology of these samples (Figure 1a,b). In the case of open porosity, the Hanks’ solution remains in contact with the DLC coating and Cr sublayer, and the degradation processes are initiated at the bottom of the pores [41]. This assumption is supported by (1) the similar breakdown potentials registered for 316L uncoated and coated with Cr/DLC (especially with Cr/0.5DLC), as determined by the properties of the passive layers formed at the 316L/Cr–electrolyte interface, primarily for Cr_2_O_3_/Cr(OH)_3_ [38]; and (2) the demonstrated porosity of the coatings of 0.59% and 0.39% for Cr/0.2DLC and Cr/0.5DLC, respectively (Table 4). Equation (2) was used to estimate porosity [31,32]:

(2) $$P=\left(\frac{R_{p/\mathrm{substrate}}}{R_{p/\mathrm{coating}}}\right)\times 10^{-\left({\Delta E}_{\mathrm{corr}}/b_{a}\right)},$$

where *R*_p/substrate_ is the polarisation resistance of 316L steel, *R*_p/coating_ is the polarisation resistance of 316L steel coated with Me/DLC coatings, and Δ*E*_corr_ is the difference between the corrosion potential values determined for the 316L and 316L/Me/DLC systems, while *b*_a_ is the slope of the tangent to the anodic branch of the Tafel curve.

The steel substrates coated with a Ti/DLC system (Figure 4) exhibited a distinct behaviour. The polarisation curves recorded for the 316L/Ti/0.2DLC and 316L/Ti/0.5DLC samples differed from those obtained for the 316L steel substrate, particularly within the anodic branch. Additionally, it was determined that the shape of the curves was significantly influenced by the thickness of the DLC coatings.

In the case of 316L/Ti/0.2DLC, despite the low porosity and continuity of the Ti/0.2DLC system, the electrolyte is able to penetrate the carbon coating into the Ti sublayer due to the presence of voids on the sample surface, as illustrated in Figure 1c. Upon reaching the corrosion potential value, a localised slow oxidation process of Ti commences. The gradual increase in oxidation products, such as TiO_2_/Ti(OH)_2_, is the cause of the absence of a stable passive region within the anodic branch. The passive layer formed on the 316L/Ti surface is found to break down at the *E*_b_ potential of 0.362 V. In contrast to the 316L reference sample, the anodic current density stabilises, most likely due to the repassivation process taking place within the pits.

The potentiodynamic polarisation tests conducted on the 316L/Ti/0.5DLC sample demonstrated its high resistance to corrosion in Hanks’ solution (Figure 4, Table 4). It can be inferred that a continuous carbon coating devoid of any other defects, with the exception of microdroplets (cf. Figure 1d), is responsible for the observed shift in the corrosion potential towards more noble values. In parallel, this sample exhibits the highest corrosion current value of the 316L/Me/DLC systems that have been tested. It is important to note that the oxidation of the Ti sublayer, which occurs at a high rate as indicated by the *i*_corr_ value, results in its almost immediate passivation. Upon exceeding the critical passivation potential, *E*_cp_ = 0.106 V, a sharp decline in anodic current density is observed. Further fluctuations are attributed to the alternating passivation and dissolution of the sublayer material [40,42]. 

**Figure 5:**
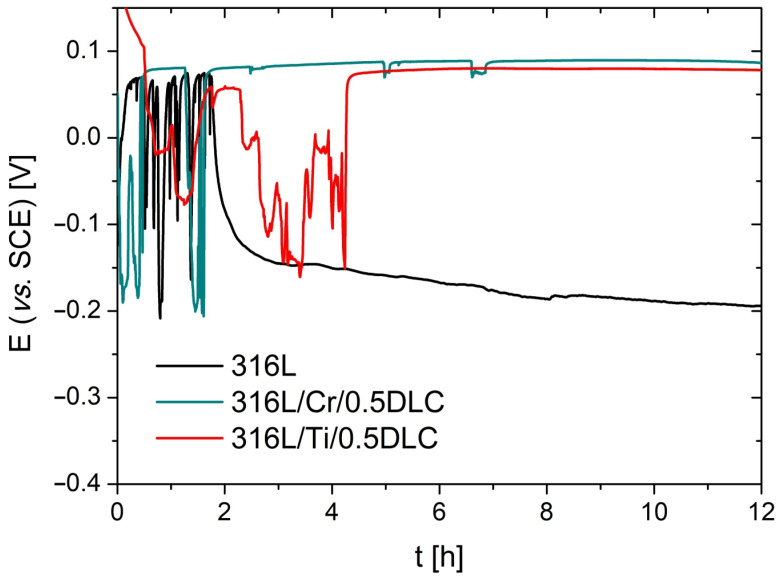
Open-circuit potentials (OCPs) recorded for the 316L, 316L/Cr/0.5DLC, and 316L/Ti/0.5DLC systems during a 12 h immersion in Hanks’ solution.

The protective properties of the Cr/0.5DLC and Ti/0.5DLC layer systems were verified by measuring the open-circuit potential during a 12 h immersion in Hanks’ solution (Figure 5). These systems exhibited higher corrosion potentials compared with Cr/0.2DLC and Ti/0.2DLC, thereby demonstrating their enhanced protective capabilities. Both systems exhibited elevated OCP values (0.089 V for 316L/Cr/0.5DLC and 0.078 V for 316L/Ti/0.5DLC) in comparison with the 316L stainless steel (−0.192 V). This phenomenon is attributed to the enhanced chemical stability of the coated substrates when subjected to the corrosive medium employed. The strong fluctuations in the OCP recorded for 316L/Ti/0.5DLC, which disappear after 5 h of immersion, confirm the occurrence of Ti passivation and transpassivation processes until stable passivity is reached. It is also noteworthy that Cr (with six outermost electrons) is nobler than titanium (with four outermost electrons). This property is likely to result in the 316L/Cr/0.5DLC system achieving a slightly higher OCP value in a shorter time [43].

**Figure 6:**
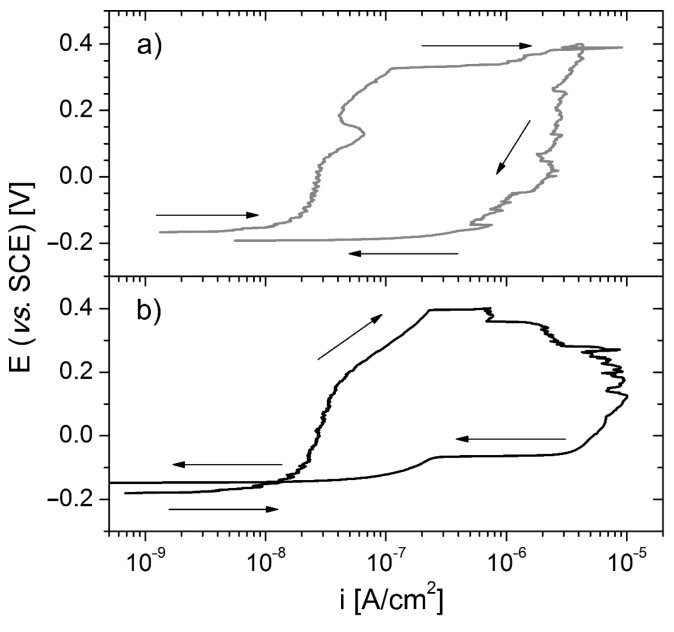
Cyclic polarisation curves for 316L/Cr/0.2DLC (**a**) and 316L/Ti/0.2DLC (**b**) recorded in Hanks’ solution. The direction of the experiments is indicated by the arrows.

**Figure 7:**
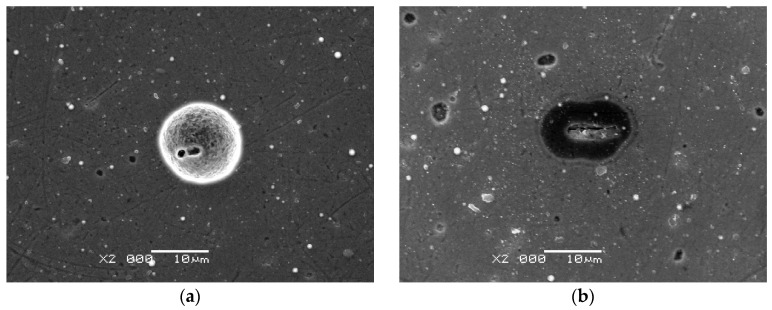
SEM images of 316L/Cr/0.2DLC (**a**) and 316L/Cr/0.5DLC (**b**) samples after corrosion tests in Hanks’ solution.

In order to gain a more profound understanding of the local corrosion (pitting) process and to ascertain the role of the sublayer in this phenomenon, cyclic potentiodynamic polarisation measurements were conducted on samples coated with thinner DLC coatings (Figure 6). In the reversed scan, a clear hysteresis loop was observed for both the 316L/Cr/0.2DLC and 316L/Ti/0.2DLC systems investigated. Hysteresis is a consequence of the destruction of the passive layer (present at the 316L/Me surface) due to the formation of pits and their further growth [44]. The aforementioned processes are terminated when a potential attains a value that is inferior to the protection potential (*E*_prot_), which represents a point of intersection between the two scans [45]. The *E*_prot_ can be identified for 316L/Ti/0.2DLC as having a value of −0.140 V. This indicates a certain capacity of the system for the repassivation of pits. Conversely, the tendency of 316L/Cr/0.2DLC towards stable pit creation was confirmed by microscopic observations made for the areas treated with Hanks’ solution. The relevant SEM image is presented in Figure 7a. In the case of 316L/Cr/0.5DLC, the presence of small corrosion centres and areas of peeling was observed (Figure 7b). It can be postulated that these originated in the vicinity of the defects of the Cr/0.5DLC system or the DLC coating. 

**Figure 8:**
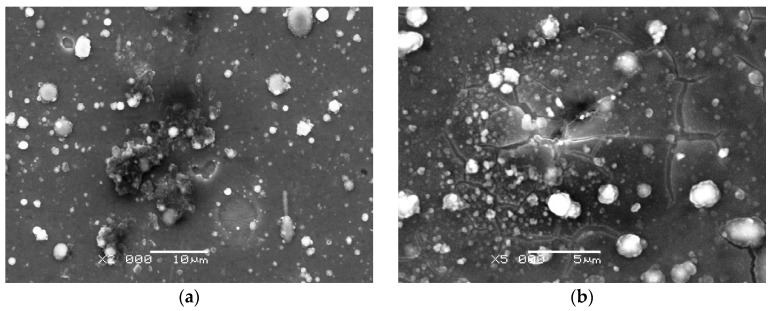
SEM images of 316L/Ti/0.2DLC (**a**) and 316L/Ti/0.5DLC (**b**) samples after corrosion tests in Hanks’ solution.

The SEM micrographs obtained for the samples with a Ti sublayer (Figure 8) revealed the presence of numerous microparticles (droplets) of deposited material, which resemble the images obtained for the pristine samples (cf. Figure 1c,d). Upon examination of the surface of 316/Ti/0.2DLC, which was exposed to Hanks’ solution, it is evident that areas of corrosion (dark areas) and coating losses with regular shapes that reveal the Ti sublayer and/or the substrate are visible. The presence of these areas may be associated with the removal of droplets that are loosely connected to the coating material. Unveiling the substrate (Cr-Ni-Mo steel) susceptible to the pitting corrosion [35,36,37] explains the presence of the hysteresis loop in the cyclic polarisation curve recorded for this sample (cf. Figure 6b). Nevertheless, no significant deterioration of the substrate was observed in this instance, as in the case of 316L/Cr/0.2DLC.

As previously stated, the Ti/0.2DLC layer system exhibited the lowest porosity value. This property can be attributed to the Ti sublayer contribution. Ti, when examined in bulk, is characterised by a stable passive state and a broad passivation plateau. In contrast, passivity occurs in a shorter potential range for Cr and 316L stainless steel [46]. In contrast to 316L/Ti/0.2DLC, the surface of 316L/Ti/0.5DLC is characterised by a few cracks only. These changes indicate that the coating is delaminating rather than exhibiting defects in the form of pits reaching the substrate material. This observation is consistent with the results of potentiodynamic polarisation measurements, which indicate the absence of a clear breakdown potential (cf. Figure 4).

In order to ascertain the adhesion of Ti/0.5DLC to the steel substrate, scratch tests were conducted, and the results were compared to those obtained for 316L/Cr/0.5DLC. The adhesion properties were characterised by the critical loads *L*_C1_ and *L*_C2_, which caused the first cohesion and adhesion damage to the coating and the film delamination within the scratch, respectively. Ti/0.5DLC exhibited reduced adhesion to the steel substrate, as evidenced by the lower critical force values observed for *L*_C1_ (6.39 ± 1.00 N) and *L*_C2_ (20.64 ± 1.63 N). In the case of Cr/0.5DLC, the critical forces were found to be 8.00 ± 0.03 N for *L*_C1_ and 28.24 ± 4.23 N for *L*_C2_. One potential explanation for the inferior adhesion of the carbon coating in the 316L/Ti/0.5DLC system is the oxidation of the Ti sublayer, which occurs with greater efficiency than in the case of the Cr sublayer. The exfoliation of the DLC coating is also evident in the SEM image obtained for the corroded sample (Figure 8b). As stated by Peng et al. [47], the formation of a thick oxide layer at the Ti surface is associated with a reduction in the interfacial toughness, which is expressed as the critical strain-energy release rate (interfacial fracture energy), *G*_ic_. In such a case, the *G*_ic_ value is found to be close to that characteristic for stainless steel and is approximately equal to 7 Jm^−2^. In accordance with the aforementioned conditions, interfacial debonding is prone to occur with relative ease. Furthermore, the incorporation of metal may result in the graphitisation of amorphous carbon coatings, thereby increasing the content of sp^2^ C bonds [48]. In their investigation of a-C coatings doped with Ti and Cr, Wang et al. [43] demonstrated that the sp^2^/sp^3^ ratio increases with increasing Ti content, in contrast to the effect observed in the case of doping with Cr. An increase in the concentration of sp^2^C bonds in the presence of Ti can facilitate the formation of a carbide phase and contribute to an increase in compressive stresses in the DLC coating while simultaneously reducing its adhesion to the substrate [49]. However, XRD studies conducted on 316L/Ti/DLC systems demonstrated that Ti carbides were not formed at the Ti/DLC interface, thereby eliminating this factor as a cause of the reduced adhesion of Ti/DLC to the 316L steel substrate. It is also noteworthy that there is a considerable discrepancy in the thermal expansion coefficients of Ti and DLC [47]. Nevertheless, this characteristic is unlikely to exert a considerable influence on the functionality of Ti/DLC systems, which are employed to safeguard the surfaces of metallic biomaterials.

## 4. Conclusions

In order to enhance the corrosion resistance of 316L to aggressive body fluids, the steel surface was modified by depositing carbon coatings (ta-C) of varying thicknesses in conjunction with the respective metallic sublayers, which were made of Cr or Ti.

A review of the results of the studies conducted on the 316L steel/sublayer/DLC coating systems revealed that the surface modification method employed contributed to an increase in steel corrosion resistance in Hanks’ solution. The deposition of the carbon layer was conducted under conditions that were conducive to the avoidance of carbide formation in the transition zone between the metallic sublayer and the DLC. This is beneficial, as carbides can have an adverse effect on the adhesion of the Me/DLC system to the steel substrate.

The combination of Ti and a DLC layer with a thickness of 0.5 µm exhibited the most effective anti-corrosion properties. For the 316L/Ti/0.5DLC system, a positive corrosion potential value was recorded. Carbon coatings with Ti sublayers exhibited lower porosity, which can be attributed to Ti’s ability to produce a stable passive state in the oxidising environment used. Nevertheless, this property was identified as one of the reasons for the low adhesion of the DLC coating to the stainless steel substrate. 

The protective properties of the Cr/DLC systems, which exhibited a higher level of discontinuity, were found to be independent of the carbon coating thickness. Conversely, the DLC coatings with a Cr sublayer demonstrated enhanced adhesion to the substrate. Therefore, the choice of the particular metallic sublayer is strictly dependent on the purpose, function, and time of use (in contact with the tissues and body fluids) of the 316L stainless steel as a metallic biomaterial. Consequently, a more detailed analysis is necessary to ascertain the mechanisms responsible for the structural and physical properties of Me/DLC coatings. In particular, the evolution of the metal/carbon transition zone, which is crucial in understanding the coating’s microstructure and composition, requires further investigation.

## Figures and Tables

**Table 1:** Chemical composition of AISI 316L stainless steel, as declared by the provider.

Element	C	Cr	Ni	Mo	Mn	S	P	Fe
Content wt.%	0.021	17.6	12.45	2.29	1.05	0.002	0.031	balance

**Table 2:** The parameters applied in the individual stages of the deposition processes.

Process Stage	Treatment Duration in One Step [s]	Pause Duration in One Step [s]	Number of Steps ^1^	*U*_bias_ [V] ^2^	*p*_Ar_ [Pa] ^3^	*I*_arc_ [A] ^4^	*I*_pulse_ [A] ^5^
Etching with Cr ions	120	60	5	−500	0.4	100	-
Deposition of Cr (or Ti) sublayer	200	60	3	−80/180	0.4	100	-
Substrate cooling to 90 °C	3600	-	1	-	-	-	-
Etching with C ions	60	60	2	−500	0.01	50	-
Etching with C ions	90	60	1	−500	0.01	50	1400
Deposition of DLC coating	30	60	6/12	0	0.01	50	1400

^1^ The number of repetitions of each stage of the process. ^2^ Substrate bias potential. ^3^ Argon pressure. ^4^ Current of the DC arc discharge. ^5^ Peak current of the pulse arc discharge.

**Table 3:** Chemical composition of the simulated body fluid–Hanks’ solution (Sigma-Aldrich).

Components	CaCl_2_ × 2H_2_O	MgSO_4_	KCl	KH_2_PO_4_	NaHCO_3_	NaCl	NaH_2_PO_4_	D-Glucose
Concentration [g/dm^3^]	0.021	17.600	12.450	2.290	1.050	0.002	0.031	balance

**Table 4:** Electrochemical parameters characterising the corrosion processes of 316L steel substrate and 316L/Me/DLC systems in Hanks’ solution.

Electrochemical Parameter	Sample				
	316L	316L/ Cr/0.2DLC	316L/ Cr/0.5DLC	316L/ Ti/0.2DLC	316L/ Ti/0.5DLC
*E*_corr_ [V]	−0.334	−0.185	−0.178	−0.154	0.061
*i*_corr_ [A/cm^2^]	26.3 × 10^−8^	1.3 × 10^−8^	0.9 × 10^−8^	0.6 × 10^−8^	11.4 × 10^−8^
*-b*_c_ [V/dec]	0.090	0.070	0.068	0.067	0.073
*b*_a_ [V/dec]	0.146	0.126	0.122	0.153	0.023
*R*_p_ [Ωcm^2^]	92 × 10^3^	1.49 × 10^6^	2.00 × 10^6^	4.70 × 10^6^	0.07 × 10^6^
*E*_b_ [V]	0.331	0.290	0.332	0.362	-
*P* [%]	-	0.59	0.39	0.11	0.26

## Data Availability

The raw data supporting the conclusions of this article will be made available by the authors on request.
